# The sound of safety: exploring the determinants of prevention intention in noisy industrial workplaces

**DOI:** 10.1186/s12889-023-17618-z

**Published:** 2024-01-04

**Authors:** Hyeon Jo, Eun-Mi Baek

**Affiliations:** 1 Headquarters, HJ Institute of Technology and Management, 71 Jungdong-ro 39, 14721 Bucheon-si, Gyeonggi-do, Republic of Korea; 2 Department of Preventive Medicine, College of Medicine, Catholic University of Korea, 222 Banpo-daero, Seocho-gu, 06591 Seoul, Republic of Korea

**Keywords:** Noise-induced hearing loss, Insomnia, Preventive behavior, Health belief model, Perceived barriers

## Abstract

Occupational noise exposure is a pervasive issue in many industries, leading to a range of health issues and sleep disturbances among workers. Additionally, there is a strong desire among these workers to prevent industrial accidents. This study, aimed at enhancing worker health and well-being, utilized a survey distributed by the Korean Confederation of Trade Unions to field workers. Data from 1285 workers were collected and analyzed using partial least squares structural equation modeling (PLS-SEM) to identify and understand the factors affecting prevention intention in noisy work environments. Our findings indicate that health problems resulting from occupational noise exposure significantly influence insomnia, perceived severity of potential accidents, perceived benefits of preventive measures, and perceived barriers. Perceived severity was significantly correlated with prevention intention, emphasizing the role of risk perception in motivating preventive behaviors. Perceived benefits were also significantly associated with prevention intention, highlighting the importance of positive outcomes in influencing workers’ behaviors. Additionally, perceived barriers showed a significant relationship with prevention intention, suggesting that overcoming these barriers is crucial in promoting preventive behaviors. Demographic factors such as gender displayed a significant association with prevention intention, while age did not. This study provides valuable insights into the multifaceted factors influencing workers’ intention to prevent industrial accidents in noisy environments, underlining the importance of comprehensive data collection tools in understanding these dynamics.

## Introduction

In this modern era, industrial noise is not merely a background element; it’s an omnipresent reality for countless workers, shaping their daily experiences and long-term health [1, 2]. The escalating issue of noise-induced hearing loss (NIHL) is starkly highlighted by the World Health Organization’s projection that by 2050, around 2.5 billion people globally — approximately 1 in 4 — will experience some form of hearing loss, with at least 700 million requiring ear and hearing care [3]. NIHL, a prevalent occupational hazard, poses a significant risk to workers in various industries [4]. The impact of excessive workplace noise extends beyond hearing damage; it impairs communication, reduces quality of life, and decreases productivity [5, 6]. Alarmingly, over 1 billion young adults face the risk of NIHL from noise exposure in both recreational settings and workplaces [7]. In the USA, for instance, one-fourth of workers are exposed to potentially harmful noise levels for a considerable part of their workday [8]. This issue is not confined to any one region; it is a globally recognized occupational problem. In Finland, NIHL was reported as the most common occupational disorder from 2012 to 2016 [9], and in Germany, it comprised 38.3% of all occupational disorders in 2019 [10]. A study spanning five European countries revealed varying incidence rates of NIHL, highlighting its recognition as an occupational disease [11]. These figures underscore the critical importance of adopting preventive behaviors and the use of hearing protection devices (HPDs) to combat NIHL [12]. These statistics serve as a sobering reminder of the need to address the comprehensive health implications of noise exposure, emphasizing a societal and economic imperative that extends far beyond the individuals directly impacted. In this vein, understanding the full spectrum of NIHL impacts is essential for fostering improved workplace health policies and practices, which could significantly enhance worker well-being and productivity.

Within the domain of occupational health, the complex interaction of elements such as health issues, sleep disorders, and factors related to prevention is crucial in influencing not only individual health but also having wider implications for society as a whole. Chronic health issues arising from workplace conditions, such as noise exposure, not only diminish individual health and productivity but also lead to increased absenteeism and reduced workplace efficiency [13, 14]. Insomnia, often linked to noise exposure, exacerbates this by heightening the risk of accidents and errors at work [15, 16]. The perception of the severity of these health risks strongly influences preventive behaviors, with a higher perceived risk driving greater engagement in safety measures [17]. However, the recognition of the benefits of preventive actions, like using hearing protection, is equally crucial in motivating these practices [18]. Conversely, perceived barriers, such as limited access to protective equipment or time constraints, significantly hinder the implementation of safety measures [19, 20]. Ultimately, the intention to engage in preventive behaviors, shaped by a combination of risk perception, benefits recognition, and barrier assessment, is a critical determinant of actual behavior change in the workplace [21]. This complex nexus of personal health, safety perceptions, and workplace practices underscores the importance of these factors in the societal and economic discourse of occupational health.

Current research on NIHL largely centers on its direct auditory consequences, frequently neglecting the broader, interconnected health effects resulting from prolonged noise exposure [22–24]. This narrow focus leaves a significant gap in understanding how these diverse health impacts, including psychological and physiological aspects, shape workers’ intentions and behaviors toward accident prevention in the workplace. Moreover, the complex interplay between exposure to workplace noise, resultant health outcomes, and consequent safety behaviors remains underexplored. There is a pressing need for a more comprehensive approach in research that not only examines the direct effects of NIHL but also explores its indirect impacts on overall worker health and safety practices. Such an approach would provide a more holistic understanding of the implications of workplace noise, thereby contributing to more effective occupational health policies and practices.

This study aims to bridge this research gap by providing a holistic framework that encompasses the multifaceted impacts of NIHL. The objective is to investigate not just the direct effects of noise on hearing but also how it affects sleep, health in daily life, and health problems in the workplace, subsequently influencing prevention behaviors. The core concerns of this research revolve around understanding the dynamics between NIHL, associated health issues, and prevention intention. The central research questions include: How do health problems of workers impact sleep and overall perceptions of preventive behavior? What is the relationship between these health impacts and workers’ prevention intentions?

The findings of this study have the potential to significantly enhance the health level of employees in noisy industrial environments. By providing empirical insights into the broader impacts of NIHL, this research can inform policymakers and workplace health practitioners, leading to more effective noise management and health promotion strategies. This study holds the potential to influence policy-making and workplace practices, ultimately benefiting the broader society by fostering safer and healthier work environments.

This paper is organized as follows: Sect. 2 presents the theoretical background, followed by the research model in Sect. 3. Section 4 details the research methodology, while Sect. 5 delves into the empirical results. Section 6 offers an in-depth discussion. Theoretical contributions and practical implications are summarized in the conclusion in Sect. 7, and the paper concludes with Sect. 8, outlining limitations and suggesting avenues for future research.

## Theoretical background

### NIHL

Occupational noise exposure is increasingly recognized as a significant factor in the emergence of hearing loss over one’s lifetime. Hong et al. [25] noted that noise exposure is a predominant contributor to NIHL, affecting an estimated 7% of the global population, as suggested by Sliwinska-Kowalska [4]. Beyond hearing impairment, NIHL has been linked to various adverse health outcomes and financial burdens. For instance, Ding et al. [26], Passchier-Vermeer and Passchier [27], and Southall et al. [28] identified a negative impact of NIHL on health, quality of life, and social well-being. Moreover, Themann and Masterson [13] discussed the extensive financial expenditures associated with NIHL.

The broader health implications of NIHL extend to an array of diseases and side effects. Deal [29] highlighted a connection between hearing loss and conditions such as dementia, hypertension, heart disease, depression, and increased rates of hospitalizations. NIHL, one of the most prevalent occupational disorders, affects a substantial portion of the workforce. Nodoushan et al. [30] estimated that over 22 million American workers are exposed to hazardous noise levels, showcasing the vast number of individuals at risk. Lie et al. [31] further illustrated that occupational noise exposure contributes significantly to hearing loss among workers, with prevalence rates ranging from 7 to 21%. This study also highlighted a disparity in incidence rates between developed and developing countries, indicating higher risks in developing nations. The prevalence of NIHL varies geographically. Kerns et al. [8] and Feder et al. [32] reported that between 12 and 19% of American workers and 15-34% of Canadian workers have experienced NIHL.

Occupations such as military, forestry, agriculture, fishing, and hunting are particularly associated with increased risks of NIHL, as noted by Lie et al. [31] and Masterson et al. [33]. To combat NIHL, it is crucial for workers in noisy environments to use protective equipment effectively. Employers play a key role in this aspect by providing guidance, setting exposure limits, and conducting regular noise monitoring and health check-ups, as emphasized by occupational safety and health administration (OSHA) [34]. These proactive measures are essential for reducing the impact of noise exposure and fostering a safer, healthier work environment.

### Impact of noise exposure in the workplace

Exposure to high levels of noise in the workplace poses a significant threat to workers’ health and well-being. Research by Burns et al. [35] and Li et al. [1] underscores the detrimental effects of such exposure, which include not only hearing loss but also insomnia and stress-related health problems. Previous studies have documented the various health consequences of noise exposure, which extend beyond auditory damage to encompass mental health issues [26, 36, 37].

The long-term impacts of noise exposure in workplace environments are far-reaching [38–40]. Halperin [41] identified a correlation between prolonged noise exposure and a decrease in workplace productivity, along with an increase in absenteeism rates. Studies collectively highlight the multifaceted nature of noise exposure’s impact on employees [42, 43]. Specifically, it was found that prolonged exposure to noise levels exceeding 85 decibels (dB) poses a significant risk to auditory health [44–46]. This risk becomes particularly pronounced when noise levels exceed 89 dB for five or more hours each week, leading to potential permanent hearing damage, as reported by Imam and Hannan [47]. Seixas et al. [48] noted that the risk of hearing loss is exacerbated by both the intensity of the noise and the duration of exposure. Basner et al. [49] delved into the psychological and social ramifications of hearing loss due to industrial noise. The study highlighted that such hearing loss can lead to increased anxiety, diminished social interactions, loneliness, sleep disturbances, concentration difficulties, depression, and an overall reduction in quality of life. These findings suggest that the impact of noise exposure in the workplace extends beyond physical health, affecting mental well-being and social functioning. This underscores the need for comprehensive workplace health policies that address both the prevention of noise exposure and its broader impacts on employee health.

### Preventive behaviors

The implementation of preventive practices, such as the use of HPDs, noise level reduction, and exposure time limitation, is crucial in preventing NIHL. Demirtaş et al. [50] emphasized the importance of preventing hearing loss due to its health, quality of life, and socioeconomic impacts. Despite the OSHA regulations introduced in 1983 to mitigate hazardous noise exposure at work [34], occupational NIHL remains a significant contributor to hearing losses. Chen et al. [51] and Sayler et al. [52] have underscored the effectiveness of these practices, particularly the significant risk reduction afforded by HPDs like earplugs and earmuffs. The relationship between HPD usage, delayed onset of hearing loss, and worker interpersonal connections was explored by Lusk et al. [53] and Olusanya et al. [54], who found that workers with perceived good hearing status were more likely to use HPDs. Hayes et al. [55] reported that among Thai workers, perceived susceptibility and severity of NIHL can predict HPD usage. Demirtaş et al. [50] and Sliwinska-Kowalska and Davis [56] noted that common HPDs such as earplugs and earmuffs can reduce noise exposure levels by 20 to 30 dB. Factors influencing HPD usage, identified by Hong et al. [25] and Melamed et al. [57], include perceived self-efficacy, noise irritation, and perceived barriers and benefits of wearing HPDs.

Despite the availability of various preventive behaviors, real-world adoption by workers remains inadequate. Kanji et al. [58] and the Who [59] observed that misconceptions and a lack of awareness about the efficacy of HPDs often lead to low adoption rates among workers. Many workers either are unaware of the permanent damage caused by loud noises [60] or underestimate the risk of irreversible hearing loss [61]. The inconvenience and communication disruption caused by HPDs are common reasons for their non-use [57, 62–64]. Several scholars have highlighted barriers to adopting preventive behaviors, such as lack of NIHL risk awareness, misconceptions about HPD effectiveness, discomfort, interference with communication, and non-enforcement of laws [65–67]. Seixas et al. [68] suggested that low adoption rates are also due to inadequate instruction on HPD usage.

Recent researchers have proposed effective strategies to prevent hearing loss. Federman and Duhon [69] and Federman et al. [70] demonstrated that proper HPD fit-testing instruction is an effective strategy. Gong et al. [71] also supported the idea that appropriate education on HPD fit-testing can help overcome barriers to preventive behaviors.

In reviewing the existing literature on NIHL, it becomes apparent that while comprehensive data exists on its prevalence and impact, there is a lack of in-depth research into the psychological and social aspects of NIHL, especially regarding the stigma and social isolation associated with hearing loss. This highlights the need for future research to focus not only on the physical aspects of NIHL but also on its broader psychosocial impacts. Additionally, the disparity in NIHL incidence rates between developed and developing countries calls for more targeted research in developing nations to identify specific occupational risks and develop culturally appropriate prevention strategies. The literature review thus points to the necessity of a more holistic approach in understanding and addressing NIHL, encompassing both physical health and socio-economic dimensions, to effectively combat this growing occupational hazard. In this vein, this study aims to fill these gaps by providing a comprehensive understanding of the factors influencing preventive behavior adoption and offering practical solutions to enhance their effectiveness.

## Research model

**Fig. 1:**
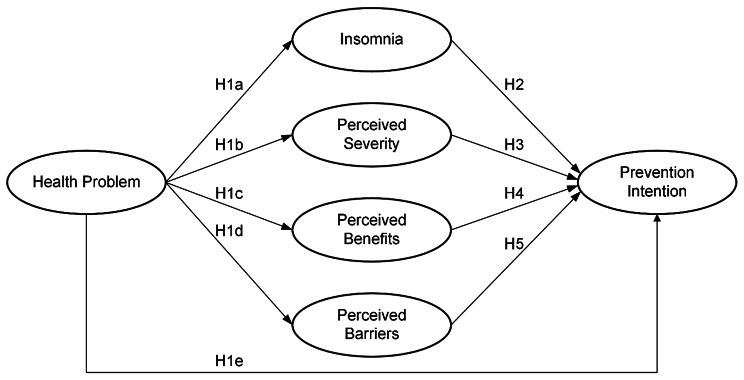
Research framework

As shown in Fig. 1, this research designates health problems as an exogenous variable. It posits that health problems affect insomnia, perceived severity, perceived benefits, and perceived barriers, as well as prevention intention. Moreover, the current study postulates that prevention intention is influenced by insomnia, perceived severity, perceived benefits, and perceived barriers.

### Health problem

Health problems can significantly impact job stress, job performance, and overall achievement, creating a multifaceted influence within the workplace [72]. Additionally, individuals with more health issues often experience poorer sleep quality [73, 74]. In noisy work environments, workers dealing with health problems may perceive their risk of hearing loss more acutely. This heightened perception often correlates with an increased desire to restore health and a stronger belief in the effectiveness of preventive behaviors [21]. Moreover, these individuals are more likely to perceive barriers to engaging in preventive actions, as their health concerns could compound the challenges or limitations faced in implementing such measures. Conversely, those experiencing health issues tend to be more inclined towards taking preventive measures against hearing loss [75]. Based on these considerations, this paper proposes the following hypotheses.

H1a. Health problem positively influences insomnia.

H1b. Health problem positively influences perceived severity.

H1c. Health problem positively influences perceived benefits.

H1d. Health problem positively influences perceived barriers.

H1e. Health problem positively influences prevention intention.

### Insomnia

Insomnia, as a diagnostic category, includes symptoms such as prolonged sleep onset, difficulty staying asleep, and the perception of unsatisfying or inadequate sleep, all of which can negatively impact daytime functioning [76]. Workplace noise has been identified as a contributing factor to insomnia [26], with studies noting its particular impact on workers [77, 78]. Workers suffering from insomnia may be more inclined to engage in hearing loss prevention behaviors to eliminate the cause of their condition. Therefore, this article proposes the following hypothesis:

H2. Insomnia positively influences prevention intention.

### Perceived severity

Perceived severity involves an individual’s subjective assessment of the seriousness or potential consequences of a particular health issue [79]. According to the Health Belief Model (HBM), perceived severity is a crucial factor influencing an individual’s decision to adopt preventive health behaviors [80, 81]. The impact of perceived severity on preventive behaviors has been established in various studies [82–84]. For an individual to take preventive action, they must view the health issue as serious enough to warrant such measures. A person who believes that noise poses a serious health risk is more likely to engage in behaviors to prevent NIHL compared to someone who perceives noise as a less serious threat. Consequently, this study hypothesizes that:

H3. Perceived severity positively influences prevention intention.

### Prevention benefits

Perceived benefits are the belief that a specific new or alternative behavior will be effective in preventing or detecting disease, maintaining health, or ameliorating the adverse effects of a health condition [85]. Within the health belief framework, perceived benefits are a vital component in understanding preventive behaviors, as they significantly influence an individual’s motivation to engage in health-promoting activities. The role of perceived benefits in driving health preventive behaviors has been explored in several studies [86, 87]. Workers who strongly believe in the benefits of actions to prevent NIHL are more likely to adhere to preventive behaviors. Thus, this research suggests that:

H4. Perceived benefit positively influences prevention intention.

### Perceived barriers

Perceived barriers represent an individual’s assessment of the obstacles and challenges to adopting health-promoting behaviors [81]. These barriers can range from financial constraints and time limitations to lack of knowledge and physical or psychological discomfort [80]. Serving as significant determinants of motivation, perceived barriers can impede an individual’s willingness or ability to undertake health-promoting actions. Research has consistently shown that perceived barriers can negatively impact the intention or actual engagement in preventive behaviors [21, 88]. Those facing higher levels of perceived barriers are less likely to adhere to activities aimed at preventing NIHL [19, 20]. In light of this, the current study hypothesizes that perceived barriers adversely affect the intention to engage in prevention practices.

H5. Perceived barriers negatively influence prevention intention.

### Control variables

In exploring health prevention behaviors, numerous studies have identified demographic factors such as gender and age as critical control variables [89–91]. Acknowledging this, our study incorporates gender and age as control variables. Additionally, considering that the level of noise exposure perceived by workers can influence their preventive behaviors, noise level is also included as a control variable in this research. These control variables are essential for providing a comprehensive understanding of the factors influencing prevention behaviors in the context of occupational health and safety.

## Research methodology

### Instrument development

To ensure the robustness of the factors considered in the research model, the survey questions were adapted from existing literature related to health beliefs, insomnia, and preventive behavior. The questionnaire items were modified to align with the specific context of NIHL. Initially, the questionnaire was developed in English by the authors and later translated into Korean by a bilingual researcher specializing in health research. To ensure accuracy, the Korean version was back-translated into English. Two experts in health studies and quantitative research thoroughly reviewed the questionnaire for its wording, structure, content, arrangement, and clarity. A pilot study involving 20 participants was conducted to assess the effectiveness of the questionnaire and to make any necessary adjustments based on the feedback received. To select participants with expertise relevant to this study, we focused on recruiting professors and researchers in the fields of public health and nursing who are affiliated with universities and university hospitals in South Korea. These professionals were specifically targeted due to their extensive knowledge and experience in areas closely related to our research topic. We disseminated information about our study to individuals working in these disciplines and recruited volunteers who expressed interest in participating. This approach ensured that the assessment of our questionnaire was conducted by individuals with a high level of expertise and a comprehensive understanding of the subject matter, thereby enhancing the validity and reliability of our research instrument. All variables, except for demographic information and frequency, were measured using a 5-point Likert scale. The measurement items for each construct can be found in Table 1.

**Table 1:** List of constructs and items

Construct	Items	Mean	Reference
Health Problem	HTP1	My health problems make it harder for me to manage job stress.	[72, 92]
	HTP2	My health problem prevents me from enjoying my job.	
	HTP3	I am not confident about completing certain tasks because of my health problem.	
Insomnia	INS1	I have difficulty falling asleep.	[93]
	INS2	I have difficulty staying asleep.	
	INS3	I wake up easily during sleep.	
Perceived Severity	PSV1	I believe that preventing noise.	[94, 95]
	PSV2	I am worried that if I have severe noise exposure, it will affect my hearing.	
	PSV3	I am concerned that my family will be affected by my noise exposure.	
Perceived Benefits	PBF1	Wearing protective equipment can prevent occupational accidents.	[85, 94]
	PBF2	Occupational accidents can be prevented by following safety rules.	
	PBF3	Wearing the right earplugs can prevent noise.	
Perceived Barriers	PBR1	I’m too busy at work to follow protective measures.	[85, 94]
	PBR2	I don’t want to be trained to wear protection.	
	PBR3	My workplace does not prioritize safety.	
Prevention Intention	PRI1	I will wear protective equipment to prevent occupational accidents.	[96] [97]
	PRI2	I will follow safety rules to prevent occupational accidents.	
	PRI3	I will receive safety training to prevent occupational accidents.	

### Data collection

The theoretical framework was validated through the collection and analysis of data obtained from an offline-based survey. The purpose of the survey in this study was to enhance the health and well-being of workers. By administering the survey, valuable insights were gained to examine and validate the relationships proposed in the theoretical framework. For this purpose, the Korean Confederation of Trade Unions distributed the online questionnaire to the field workers. The data collection for this study was conducted from July 8th to October 30th, 2022. Using the health management network of the workplace, the researchers obtained prior approval from health managers. The managers conducted the survey. The introductory section of the survey provided a clear explanation of the study’s purpose and its intent for academic publication. Informed consent was obtained from all individual participants who were included in the study, ensuring their voluntary participation. Only those who explicitly agreed to have their responses published were included in the survey. To mitigate attention constraints inherent in the online survey process, the study employed the use of negative constructs as a strategic approach and attention trap issues (unrelated to our research). For workers in the cooking industry, the survey was carried out by distributing questionnaires directly to participants who understood the purpose, method, procedures, anonymity, and the possibility of withdrawal during the research without any disadvantages. The completion of the questionnaire required approximately 10 min of participants’ time. The collected data was encrypted and stored on the researcher’s personal computer. After the completion of the study, the data was securely disposed of. Following the removal of insincere responses through data filtering, a total of 1285 responses were retained for further analysis.

Table 2 presents the profile of the respondents in terms of their demographics. The table includes information on gender, age, industry, and noise level. In terms of gender, 431 respondents (33.5%) identified as male, while 852 respondents (66.3%) identified as female. A small number of respondents (2, 0.2%) did not provide their gender information. The age distribution of the respondents is as follows: 1 respondent (0.1%) in their 10s, 59 respondents (4.6%) in their 20s, 214 respondents (16.7%) in their 30s, 426 respondents (33.2%) in their 40s, 551 respondents (42.9%) in their 50s, and 30 respondents (2.3%) in their 60s. A few respondents (4, 0.3%) did not disclose their age. Regarding the industry, 340 respondents (26.5%) worked in the manufacturing sector, 19 respondents (1.5%) were in the construction industry, 5 respondents (0.4%) were involved in shipping, 771 respondents (60.0%) were in the cooking industry, and 150 respondents (11.7%) were categorized as “other” indicating other industries. The noise level, measured in dB, varied among the respondents. A total of 70 respondents (5.4%) reported a noise level of 60 or less dB, 106 respondents (8.2%) reported a noise level between 60 and 69 dB, 115 respondents (8.9%) reported a noise level between 70 and 79 dB, 127 respondents (9.9%) reported a noise level between 80 and 89 dB, 69 respondents (5.4%) reported a noise level between 90 and 99 dB, 69 respondents (5.4%) reported a noise level of 100 or more dB, and the majority of respondents (729, 56.7%) were unsure or did not provide information about the noise level.

**Table 2:** Profile of the respondents

Demographics	Item	Subjects (*N* = 1285)	
		Frequency	Percentage (%)
Gender	Male	431	33.5
	Female	852	66.3
	Not respond	2	0.2
Age	10s	1	0.1
	20s	59	4.6
	30s	214	16.7
	40s	426	33.2
	50s	551	42.9
	60s	30	2.3
	Not respond	4	0.3
Industry	Manufacturing	340	26.5
	Construction	19	1.5
	Shipping	5	0.4
	Cooking	771	60.0
	Other	150	11.7
Noise Level (dB)	60 or less	70	5.4
	60–69	106	8.2
	70–79	115	8.9
	80–89	127	9.9
	90–99	69	5.4
	100 or more	69	5.4
	Don’t Know	729	56.7

## Research results

Partial Least Squares Structural Equation Modeling (PLS-SEM) was applied in this study because it is particularly suited for exploratory research and theory development, where the primary goal is prediction and explanation of target constructs. PLS-SEM allows for the modeling of complex relationships between observed and latent variables, even when data are non-normal, thus making it a powerful tool for the analysis of complex structural models [98].

### Common method bias

The issue of common method bias was considered due to the self-report nature of our data collection method. Common method bias refers to the spurious variance that is attributable to the measurement method rather than to the constructs the measures represent [99]. When data for both predictor and criterion variables are collected from the same individual at the same time, the correlations between variables can be artificially inflated due to common method bias, potentially leading to misleading results. Several procedural and statistical remedies were implemented in this study to mitigate the risk of common method bias. Procedurally, the survey questions were designed to be clear and concise, and anonymity of responses was assured to encourage honest reporting and to minimize any potential social desirability bias [100]. Statistically, Harman’s single-factor test was conducted to detect the presence of common method bias. This involves performing a factor analysis on all the items in the questionnaire to see if a single factor emerges or if one general factor accounts for the majority of the covariance among the measures [99]. The results showed that the percent of the variance of a single construct was 24.060, indicating that common method bias was not a serious concern in our data.

### Measurement model

The measurement model was assessed using a two-step process, beginning with an examination of the reliability and validity of the scale items, followed by an evaluation of the discriminant validity of the constructs.

Table 3 reports the reliability and convergent validity of the measurement scales. The factor loadings of all items exceeded the threshold of 0.70, which supports the item reliability [98]. The values for Cronbach’s alpha, composite reliability (CR), and average variance extracted (AVE) for each construct all met or surpassed their respective criteria (Cronbach’s alpha and CR > 0.70, AVE > 0.50), confirming the reliability and convergent validity of the measures [98].

**Table 3:** Scale reliability

Construct	Items	Mean	St. Dev.	Factor Loading	Cronbach’s Alpha	CR	AVE
Health Problem	HTP1	3.014	1.104	0.832	0.806	0.886	0.721
	HTP2	2.947	1.174	0.880			
	HTP3	2.399	1.087	0.834			
Insomnia	INS1	2.119	1.043	0.890	0.894	0.934	0.826
	INS2	2.155	1.063	0.939			
	INS3	2.407	1.101	0.896			
Perceived Severity	PSV1	3.988	0.833	0.814	0.808	0.883	0.716
	PSV2	3.554	1.103	0.865			
	PSV3	3.529	1.122	0.858			
Perceived Benefits	PBF1	3.311	0.969	0.735	0.776	0.868	0.687
	PBF2	3.933	0.853	0.876			
	PBF3	3.693	0.854	0.868			
Perceived Barrier	PBR1	2.713	1.604	0.742	0.624	0.799	0.570
	PBR2	2.293	0.976	0.734			
	PBR3	2.514	1.133	0.787			
Prevention Intention	PRI1	3.900	0.857	0.856	0.873	0.922	0.798
	PRI2	4.154	0.731	0.934			
	PRI3	4.232	0.717	0.888			

Correlation analysis was used to assess the relationships among the research variables. As shown in Table 4, the constructs’ square root of the AVE was greater than their correlation coefficients with other constructs, indicating good discriminant validity [101].

**Table 4:** Correlation of the research variables

Constructs	1	2	3	4	5	6
1. Health Problem	0.849					
2. Insomnia	0.333	0.909				
3. Perceived Severity	0.211	0.165	0.846			
4. Perceived Benefits	-0.099	-0.077	0.304	0.829		
5. Perceived Barriers	0.340	0.254	0.146	-0.110	0.755	
6. Prevention Intention	-0.140	-0.086	0.317	0.478	-0.338	0.893

The Heterotrait-Monotrait ratio (HTMT) was utilized as an additional test for discriminant validity. The values, as shown in Table 5, were below the conservative threshold of 0.85 [102], further supporting the discriminant validity of the constructs.

**Table 5:** HTMT

Constructs	1	2	3	4	5	6
1. Health Problem						
2. Insomnia	0.390					
3. Perceived Severity	0.279	0.199				
4. Perceived Benefits	0.124	0.094	0.358			
5. Perceived Barriers	0.472	0.335	0.283	0.154		
6. Prevention Intention	0.168	0.097	0.343	0.563	0.461	

In summary, the measures used in the study demonstrated satisfactory psychometric properties, which lends confidence to the subsequent analysis of the structural model.

### Structural model

**Fig. 2:**
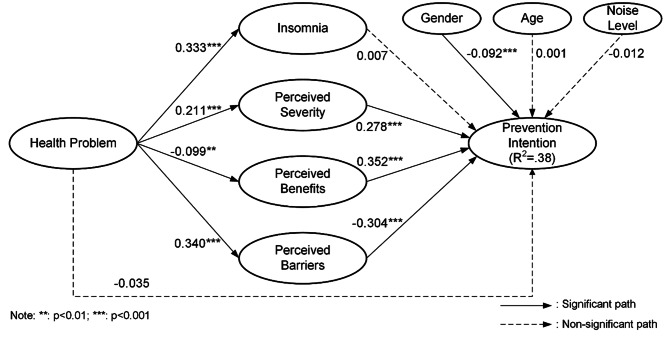
Analysis results (PLS algorithm)

After confirming the validity of the measurement model, the next step involved examining the structural model. To assess the structural model, a bootstrapping procedure with 5000 bootstrap samples was performed using PLS-SEM. This procedure allowed us to estimate the precision of the path coefficients and determine their significance, thereby validating the hypotheses proposed in this study. The resulting path coefficients, standard errors, *t*-values, and *p*-values were analyzed to gain insights into the hypothesized relationships. Significance was determined by evaluating the *t*-values against a significance level of 0.05. The analysis results of the PLS algorithm are presented in Fig. 2, providing an overview of the findings.

In keeping with expectations, health problems have a significant positive impact on insomnia (*b =* 0.333, *t =* 12.691) and perceived severity (*b =* 0.211, *t =* 6.386), strongly supporting hypotheses H1a and H1b. Contrary to expectations, health problems have a negative effect on perceived benefits (*b=*-0.099, *t =* 3.220), which does not support H1c. Consistent with the hypothesis, health problems have a positive correlation with perceived barriers (*b =* 0.340, *t =* 11.575), supporting H1d. In contrast to the hypothesis, health problems do not affect prevention intention (*b=*-0.035, *t =* 1.376), failing to support H1e. Unexpectedly, insomnia does not influence prevention intention (*b =* 0.007, *t =* 0.284), failing to support H2. As hypothesized, perceived severity has a significant correlation with prevention intention (*b =* 0.278, *t =* 9.207), strongly supporting H3. In line with the hypothesis, perceived benefits are significantly associated with prevention intention (*b =* 0.352, *t =* 12.35), strongly supporting H4. As predicted, perceived barriers negatively impact prevention intention (*b=*-0.304, *t =* 8.991), strongly supporting H5. Consistent with predictions, gender has a significant correlation with prevention intention (*b=*-0.092, *t =* 3.382). Unexpectedly, age (*b =* 0.001, *t =* 0.064) and noise level (*b=*-0.012, *t =* 0.54) do not significantly affect prevention intention. Overall, the research model accounted for approximately 38.0% of the variance in prevention intention. Table 6 describes the results of the structural model.

**Table 6:** The results of structural model

H	Cause	Effect	Coefficient	T-value	***P***-value	Hypothesis
H1a	Health Problem	Insomnia	0.333	12.691	0.000	Supported
H1b	Health Problem	Perceived severity	0.211	6.386	0.000	Supported
H1c	Health Problem	Perceived Benefits	-0.099	3.220	0.001	Not Supported
H1d	Health Problem	Perceived Barriers	0.340	11.575	0.000	Supported
H1e	Health Problem	Prevention Intention	-0.035	1.376	0.170	Not Supported
H2	Insomnia	Prevention Intention	0.007	0.284	0.776	Not Supported
H3	Perceived Severity	Prevention Intention	0.278	9.207	0.000	Supported
H4	Prevention Benefits	Prevention Intention	0.352	12.350	0.000	Supported
H5	Perceived Barriers	Prevention Intention	-0.304	8.991	0.000	Supported
CV	Gender	Prevention Intention	-0.092	3.382	0.001	Supported
CV	Age	Prevention Intention	0.001	0.064	0.949	Not Supported
CV	Noise Level	Prevention Intention	-0.012	0.540	0.589	Not Supported

Note: CV stands for control variables

## Discussion

This study aimed to examine the factors influencing prevention intention in the context of NIHL by considering health problems, components of the health belief model, and control variables.

This research revealed significant insights into the relationships between health problems, insomnia, perceived severity, perceived benefits, perceived barriers, and workers’ prevention intentions in the context of noise-induced environments. Our findings indicate that health problems significantly contribute to insomnia (H1a) and perceived severity (H1b), but interestingly, they negatively impact perceived benefits (H1c). Health problems affects perceived barriers (H1d). However, the direct relationship between health problems and prevention intention (H1e) was not supported. Additionally, our study did not find a significant direct effect of insomnia on prevention intention (H2). Contrarily, perceived severity (H3) and perceived benefits (H4) significantly influenced prevention intention, as did perceived barriers (H5). Gender was found to have a significant effect on prevention intention, while age and noise level did not significantly affect prevention intention.

The objective of this research was to explore the dynamics between health problems due to noise exposure and their subsequent impact on workers’ preventive intentions. The findings have provided a clearer understanding of these relationships, particularly the significant role of perceived severity and benefits in motivating preventive behaviors in noisy work environments.

This study significantly advances the understanding of how health problems resulting from workplace noise exposure influence workers’ attitudes and behaviors towards preventive measures. Our findings align with and extend existing literature on the complexity of preventive behavior in occupational settings [103–105], offering a more nuanced view of the interdependencies among health issues, perceptions, and preventive actions.

One of our key discoveries is the direct impact of health problems on insomnia and perceived severity, accompanied by a surprising decrease in the perceived benefits of preventive actions. The empirical relationship between health problems and insomnia could be attributed to the fact that workers with more health problems get less sleep. This aligns with observations in past research [73, 74] and is likely due to mental and physical fatigue interfering with sleep quality. The significant correlation between health problems and perceived severity indicates that workers with more health issues are more cognizant and concerned about NIHL. This heightened awareness likely arises from their increased vulnerability to various health risks and hazards. It underscores the importance of targeted health communication strategies in workplaces to raise awareness and encourage preventive behaviors among all workers, regardless of their current health status. The negative effects of health problems on perceived benefits suggest that workers experiencing health issues might underestimate the benefits of prevention. This finding adds a new dimension to the development of workplace health interventions. This underestimation may stem from a lack of awareness or the overwhelming nature of their current health issues, overshadowing the long-term advantages of preventive measures.

Our research supports the positive relationship between perceived severity and prevention intention, in line with the health belief model. This model suggests that the perceived seriousness of a health risk is a powerful motivator for individuals to adopt protective behaviors [82–84]. The findings corroborate previous studies [86, 87], indicating that individuals who perceive higher severity in workplace hazards are more likely to engage proactively in preventive actions. Additionally, perceived benefits significantly influence prevention intentions. Workers who believe that preventive actions, such as wearing protective gear or following safety practices, will effectively reduce their risk of NIHL are more inclined to adopt such measures. This highlights the need for clear and effective communication about the benefits of preventive behaviors in the workplace. Notably, perceived barriers emerged as a significant hindrance to prevention intention, in line with existing literature [21, 88]. This finding indicates that when workers face practical difficulties or perceive preventive measures as inconvenient, their willingness to engage in these behaviors decreases. Addressing these barriers, whether they be time constraints, lack of resources, or workplace culture, is crucial for enhancing preventive intentions.

Concerning control variables, the significant influence of gender on prevention intention is particularly revealing. This suggests a gender-specific approach in addressing workplace safety, considering the different exposure levels and perceptions between men and women in industrial settings, particularly in South Korea.

In summary, this study provides valuable insights into how health problems and perceptions about noise exposure and its consequences shape preventive behaviors in the workplace. It underscores the importance of comprehensive approaches that address not only the direct impacts of NIHL but also the broader, indirect effects on worker health and safety. The findings have significant implications for designing effective workplace health and safety policies, programs, and interventions.

## Conclusion

### Implications for researchers

This section delves into the theoretical contributions of our study, highlighting its significance within the broader context of occupational health research.

The first key contribution of our research lies in its comprehensive examination of the effects of NIHL on various facets of worker health and behavior. Previous studies have predominantly focused on the immediate auditory impacts of NIHL [22–24]. Our study, however, extends this understanding by exploring how health problems related to NIHL influence workers’ sleep quality, perceived severity, and perceived benefits of preventive actions. This broader perspective is crucial for developing more effective health interventions in noisy work environments. By highlighting these broader impacts, our research encourages future studies to adopt a more holistic approach when examining the consequences of workplace noise exposure.

Our second contribution is the examination of the relationship between health problems and prevention intention. While existing literature has often focused on the direct impact of workplace hazards on prevention behaviors [106–108], our study reveals a more complex relationship. Health problems were found to indirectly affect prevention intention through perceived severity and benefits, rather than directly. This finding suggests that workers’ health perceptions play a critical role in shaping their preventive behaviors, a notion that is relatively underexplored in current literature. This insight provides a new avenue for occupational health researchers to explore, particularly in the context of designing interventions that aim to enhance workers’ perception of the severity and benefits of preventive actions.

The third significant contribution of our study is the exploration of perceived barriers as a critical factor in prevention intention. Previous research has acknowledged the importance of perceived barriers in health behavior models [109–111]. Our study builds upon this by specifically linking these barriers to NIHL prevention in the workplace. We found that practical challenges and perceptions of inconvenience significantly deter workers from engaging in preventive behaviors. This finding has substantial implications for workplace policy and practice, suggesting that reducing perceived barriers could significantly improve preventive behaviors among workers. Future research could explore strategies to minimize these barriers, thereby enhancing the effectiveness of workplace safety programs.

Finally, our research underscores the importance of gender differences in 7 prevention intention. While the impact of gender on preventive health behaviors has been noted in previous studies [112–114], our study adds to this literature by focusing on noise-related workplace settings. We observed that men showed a higher intention to prevent occupational accidents, a finding that has significant implications for workplace safety policies. It suggests that gender-specific strategies may be needed to effectively address prevention in diverse workplace environments. This aspect of our research invites scholars to further investigate gender dynamics in occupational health behaviors, particularly in relation to noise exposure and prevention strategies.

### Managerial implications

This study’s findings offer several practical implications that can significantly influence workplace health management, policy-making, and operational practices.

The first practical implication relates to the development of targeted health interventions in noisy work environments. Our research has shown that health problems arising from NIHL not only affect physical health but also impact mental well-being, manifesting as increased insomnia and altered perceptions of severity and benefits of preventive actions. For workplace health managers, this suggests the need for comprehensive health programs that address both auditory and non-auditory effects of noise exposure. Implementing strategies that include regular hearing tests, providing education on the importance of hearing protection, and interventions aimed at improving sleep quality can be instrumental in mitigating the broader impacts of NIHL [25, 115].

Secondly, our findings highlight the importance of raising awareness about the severity of NIHL and its broader implications. This is crucial for policy-makers who are in a position to influence workplace safety standards and regulations. The study suggests that enhancing workers’ understanding of the severity of NIHL can significantly improve their intention to adopt preventive measures. Policies that mandate training sessions, workshops, and awareness campaigns about the risks associated with workplace noise and the benefits of preventive practices can lead to more proactive safety behaviors among workers [116].

Furthermore, the study emphasizes the critical need to tackle perceived barriers that impede the adoption of safety measures in noisy work environments. For workplace operators and managers, this involves a proactive approach to identify and mitigate factors that hinder safety compliance [117]. Key strategies could include streamlining the process for obtaining protective gear to ensure it is readily accessible and user-friendly. Additionally, redesigning workspaces to reduce noise levels, possibly through the installation of sound-absorbing materials or the reconfiguration of machinery and equipment, can be an effective measure. Moreover, implementing more flexible and worker-friendly schedules can significantly decrease the burden of adhering to preventive practices. These concerted efforts to address barriers can not only foster a culture of safety but also lead to a tangible decrease in workplace accidents related to NIHL, enhancing overall worker safety and well-being.

Lastly, this study underscores the crucial role of individual responsibility in maintaining occupational safety, particularly in environments with noise exposure. Workers are the front line of defense against noise-induced health issues and should be motivated to engage actively in safety training programs. These programs provide vital knowledge and skills needed to navigate noisy work environments safely. Compliance with safety regulations and consistent use of protective equipment, such as earplugs or earmuffs, is essential in preventing NIHL and related health problems [118, 119]. Specifically, to preventNIHL, it is recommended to use personalized earplugs rather than generic earplugs [120, 121]. Moreover, workers should feel empowered to express concerns about workplace safety. Creating channels for open communication where workers can report potential hazards or suggest improvements can significantly enhance the overall safety culture. This empowerment not only contributes to a safer work environment but also fosters a sense of ownership and responsibility among workers. The study highlights the importance of preventive actions, extending beyond the preservation of hearing health to encompass overall well-being. Workers should recognize that their actions have a profound impact on their health and safety, and by taking proactive measures, they contribute to a healthier, more productive work environment. This recognition is fundamental in cultivating a workplace where safety is a shared responsibility and a collective goal.

In conclusion, the practical implications of this study extend across various aspects of workplace safety and health management, offering valuable insights and action points for policy-makers, workplace health managers, operators, and workers. By implementing these suggestions, the risk of NIHL and its associated health issues can be significantly mitigated, leading to safer and healthier working environments.

## Limitation and further research

While this study provides valuable insights into the impacts of NIHL on workplace behavior, it is important to acknowledge its limitations to contextualize the findings appropriately. One significant limitation is the cross-sectional nature of the study, which restricts the ability to establish causality or track changes over time. A longitudinal approach would offer a more comprehensive understanding of the dynamics and evolution of workers’ perceptions and behaviors in response to noise exposure. Additionally, the study did not fully explore the effect of potential confounding variables that might influence the relationships between health problems, insomnia, perceived severity, benefits, barriers, and prevention intention. Factors such as personal health history, workplace culture, and individual coping mechanisms could play a significant role in shaping these relationships. Future research should consider employing a longitudinal design to observe how attitudes and behaviors evolve over time in response to workplace noise exposure. Investigating the role of confounding variables could also provide deeper insights. Furthermore, expanding the scope to include qualitative assessments could offer a richer, more nuanced understanding of the subjective experiences of workers dealing with noise-induced health issues. These approaches would contribute significantly to the development of more targeted and effective workplace interventions and policies.

## Data Availability

The data used in this study are available from the corresponding authors upon reasonable request.
